# Deciphering Amyloid Fibril Formation through Protein Concentration by Optical Trapping

**DOI:** 10.1021/jacsau.5c01029

**Published:** 2025-10-15

**Authors:** Teruki Sugiyama, Shu-Ting Weng, Tien Chen, Tsuyoshi Mashima, Shun Hirota

**Affiliations:** † Department of Applied Chemistry and Center for Emergent Functional Matter Science, 34914 National Yang Ming Chiao Tung University, No. 1001, Daxue Rd. East Dist, Hsinchu 300093, Taiwan; ‡ Division of Materials Science, Graduate School of Science and Technology, 12708 Nara Institute of Science and Technology, 8916-5 Takayama-Cho, Ikoma, Nara 630-0192, Japan; § Medilux Research Center, Nara Institute of Science and Technology, 8916-5 Takayama-Cho, Ikoma, Nara 630-0192, Japan

**Keywords:** amyloid fibril formation, apoferritin, optical trapping, protein aggregation, pH control

## Abstract

Optical trapping, combined with time-lapse transmission and fluorescence imaging, enables precise real-time observation of protein concentration dynamics during amyloid fibril formation. Condensates form at the laser focus within 30 min and grow to ∼1.2 μm in diameter regardless of pD by optical trapping of apoferritin (Fer8). Initial trapping efficiency is higher at pD 8.4 (stable 24-mer) than under acidic conditions (pD 1.5 and 2.0; subunit dimer), while acidic solutions show a pronounced second-phase concentration increase after ∼72 min, ultimately far exceeding the concentration in pD 8.4. However, the second-phase surge is absent at pD 3.0, despite evidence of amyloid formation (ThT fluorescence and transmission electron microscopy (TEM)), likely due to slower elongation presumably owing to higher protein stability than at lower pD. Quantitative analysis of Fer8 subunit concentration reveals a critical concentration (0.53–0.63 mM) for the rapid ThT fluorescence increase onset for all conditions at pD 1.5–3.0. This moderate concentration, combined with secondary structure observations, suggests that optical trapping facilitates specific alignment of Fer8 molecules beyond simple concentration. These findings highlight optical trapping’s power to dissect the pD-dependent interplay between protein structure, nucleation, and amyloid fibril elongation, providing insights into the early stages of amyloid fibril formation.

## Graphical abstract

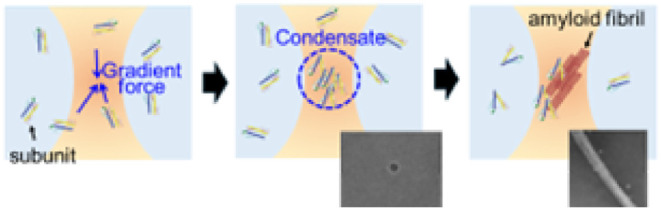

## Introduction

Amyloid fibrils are insoluble aggregates of misfolded proteins implicated in numerous debilitating diseases,^1,2^ including Alzheimer’s,^3^ Parkinson’s,^4^ and type II diabetes.^5^ While amyloid fibrils are known to form a common cross-β fold,^5,6^ understanding the intricate mechanisms, involving nucleation, elongation, and saturation phases, remains a significant challenge,^4,7,8^ Particularly, the initial nucleation phase of amyloid fibril formation occurs randomly at unpredictable times and locations, making its real-time observation and detailed analysis and prediction of its initiation site difficult.^9–11^ The complex process of amyloid formation, initiated by partial protein denaturation and the transient formation of unstable intermediates, is still not fully elucidated.^12–14^ On the other hand, amyloid fibrils are also used to create functional materials,^15–17^ making the method of controlling amyloid fibril formation a vital tool.

Optical trapping,^18,19^ a powerful method utilizing a tightly focused laser beam for noncontact trapping and manipulation of nano- and microscale particles, has emerged as a valuable tool in amyloid fibril formation research.^20–22^ The fundamental principle relies on transferring momentum from photons to the target object, generating optical forces. The Rayleigh scattering regime is applicable for nanoscale targets such as the apoferritin (Fer8) studied here. In this regime, the dominant “gradient force,” proportional to the spatial gradient of the laser intensity and the polarizability of the targets, pulls particles toward the focal point, the region of highest laser intensity. Meanwhile, the “scattering force,” due to momentum transfer from scattered photons, acts in the direction of light propagation but becomes negligible for small targets. Detailed theoretical treatment of optical trapping is presented in SI1. This method enables three-dimensional trapping and precise control of micro targets in solution. Critically for amyloid studies, optical trapping allows for detecting changes in the localized concentration of proteins within the laser focus, facilitating in situ spectroscopic measurements. This capability for spatiotemporal control and real-time observation of amyloid fibril formation is a pivotal feature of optical trapping research, promising significant contributions to biophysics, drug discovery, and beyond.

Our previous work has successfully demonstrated spatiotemporal control and detailed observation of amyloid fibril formation in a monomeric heme protein like cytochrome *c* (cyt *c*) using optical trapping.^20,21^ This study focuses on Fer8, a spherical cage-like protein composed of 24 subunits that exhibits a fundamental 4-helix bundle structure, as a model protein to study concentration dynamics during amyloid formation. Ferritin plays a crucial role in iron storage and release.^23,24^ Its confined space has also encapsulated various organic and inorganic compounds.^25–28^ The 24-mer structure of Fer8 is known to undergo significant changes in response to pH variations. Specifically, Fer8 dissociates into subunit dimers under acidic conditions^29^ and forms amyloid fibrils under acidic conditions.^30,31^ We previously showed that hydrogels are formed from concentrated Fer8 solutions by acid denaturation and subsequent neutralization.^32^ Despite the compelling nature of Fer8 for amyloid research, a comprehensive real-time investigation into protein concentration dynamics during amyloid fibril nucleation and elongation has not been conducted, primarily due to the absence of suitable measurement methods. To bridge this gap, we herein aim to comprehensively investigate Fer8 amyloid fibril formation using optical trapping under various acidic (pD) conditions by monitoring protein concentration. This novel approach will allow us to precisely elucidate the intricate effects of pH (pD) and protein concentration on the kinetics and mechanisms of amyloid fibril formation.

## Results and Discussion

### Optical Trapping and pD-Dependent Concentration Dynamics of Fer8

This section details the precise observation of trapping and concentration dynamics of Fer8 using optical trapping, comparing the condensation behaviors under different pD conditions (Supporting Information, Experimental Section/Methods section). Experiments were performed in D_2_O solutions to avoid absorption of the trapping beam by H_2_O and the subsequent temperature increase (Figure S1). This observation allows for initiating and monitoring fibril formation at a defined location, addressing the challenge of unpredictable nucleation sites. Time-lapse transmission and Dye-Fer8 fluorescence images were acquired in solutions at pD 2.0 (acidic, Fer8 in subunit dimer) and pD 8.4 (mildly alkaline, Fer8 in 24-mer). These dynamic condensation behaviors are shown in Figure 1. Results for pD 1.5 and 3.0 are presented in Figure S2. Dye-Fer8, a fluorescently labeled Fer8, enabled real-time visualization and quantitative estimation of local Fer8 concentration based on its fluorescence intensity. Given the extremely low volume ratio of Dye-Fer8 (1/800th of Fer8), its influence on the optical trapping behavior of Fer8 is considered negligible. As early as 30 min after laser irradiation commenced, the formation of condensates was observed at the laser focus in both pD 2.0 and pD 8.4 solutions. After 75 min of laser irradiation, the formed condensate grew to approximately 1.2 ± 0.1 μm in diameter, independent of the solution’s pD. However, significant differences in condensation dynamics were evident depending on the solution’s pD. In the pD 8.4 solution, the transmission rate of the condensate decreased more rapidly, and the increase in Dye-Fer8 fluorescence intensity was slightly faster than in acidic solutions (up to 60 min of laser irradiation). Although the fluorescence images in the initial stages may appear visually similar, this observation is supported by a detailed quantitative analysis, which is presented later. These results suggest a higher optical trapping efficiency in pD 8.4 solution. This is likely attributable to Fer8 maintaining its stable 24-mer cage structure at pD 8.4, whereas it dissociates into subunit dimers under acidic conditions.^29^ Intriguingly, after 75 min of laser irradiation, the Dye-Fer8 fluorescence intensity in the pD 2.0 solution significantly increased, ultimately far exceeding that in the pD 8.4 solution. This phenomenon indicates that prolonged laser irradiation at pD 2.0 led to the formation of a high-concentration region of Fer8 around the laser focus. A similar trend was observed for pD 1.5, another acidic solution, but not for pD 3.0. It has been reported that Fer8 dissociates into subunit dimers at pH 2.0 but forms a caged 24-mer above pH 2.5.^29^ Thus, the increase in fluorescence intensity, and consequently the local concentration dynamics, was pD-dependent, i.e., assembly dissociation dependent. The correlation between the structural robustness, trapping efficiency, and its impact on amyloid fibril formation will be discussed below.

**Figure 1:**
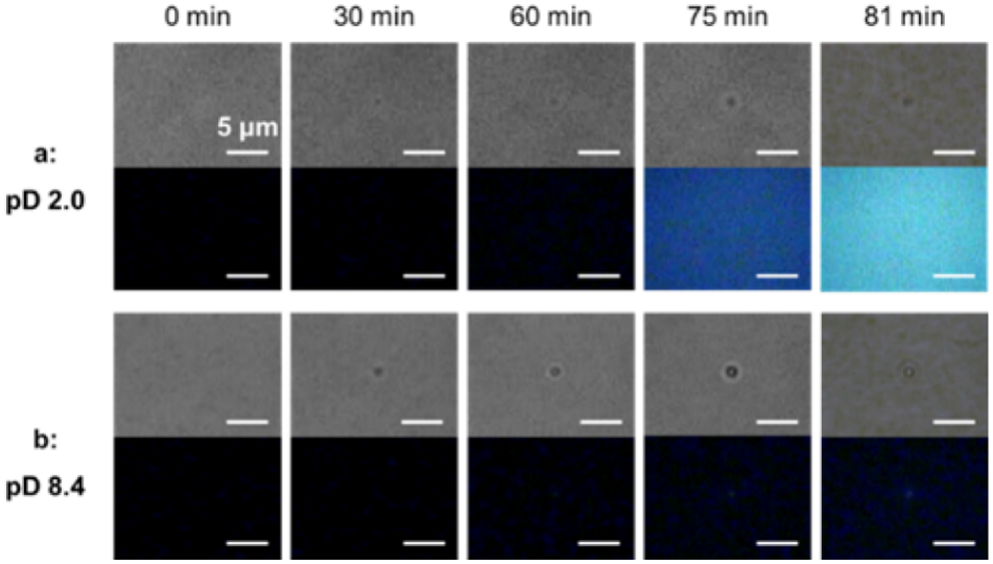
Optical trapping and localized concentration dynamics of apoferritin (Fer8) in solutions of varying pD. Time-lapse transmission (top row) and corresponding Dye-Fer8 fluorescence (bottom row) images illustrating Fer8 condensate formation and growth at (a) pD 2.0 and (b) pD 8.4.

While the gradient force is expected to result in the highest protein concentration precisely at the laser focus, the Dye-Fer8 fluorescence images at later time points (75 and 81 min) for pD 2.0 (Figure 1a) show a broader, more uniformly bright area rather than a single, highly localized bright spot. This observation suggests that the concentrated Fer8 at the focus increases in density and forms larger, more extensive condensates or aggregates that expand outward from the initial laser focus. This outward spreading of highly concentrated regions has been a phenomenon frequently observed in optical trapping experiments,^33,34^ particularly when significant condensation occurs without forming amyloid fibrils, as in the case of cyt *c*.^20,21^ The widespread brightness visually represents the increased mass and volume occupied by these growing protein assemblies. Furthermore, despite the broader appearance, transmission light measurements confirmed that the lowest transmission rate, indicative of the highest local concentration, consistently occurred at the center of the trapped region. At these extremely high local concentrations, the Dye-Fer8 fluorescence signal in the most concentrated central part might experience quenching, contributing to the more uniform brightness observed across the condensate rather than a sharply defined brightest point.

### Quantifying Local Fer8 Concentration Changes with pD and Time

Figure 2 illustrates the temporal changes in relative Fer8 concentration at the laser focus, estimated from Dye-Fer8 fluorescence intensity, for solutions at pD 2.0 and pD 8.4. The fluorescence intensity was collected from the focal region of the laser using a spectrometer with a 200 μm pinhole, ensuring a well-defined measurement area. The estimation of protein concentration was based on a calibration procedure detailed in Figure S4. It is reported that Fer8 dissociates into subunit dimers at pH 2.0.^29^ Therefore, comparing the time-dependent Fer8 concentration changes under mildly alkaline and acidic conditions allowed us to elucidate the influence of Fer8’s assembled structural state on its trapping efficiency and subsequent amyloid formation. In both pD conditions, the relative Fer8 concentration showed a nonlinear increase with laser irradiation time, yet distinct patterns of increase were observed. For the pD 8.4 solution, the relative Fer8 concentration increased relatively quickly from the onset of laser irradiation, suggesting a relatively higher trapping efficiency at the initial stage (as shown in the magnified view of Figure 2). This observation supports the results described previously regarding condensation dynamics. Conversely, in the pD 2.0 solution, the relative Fer8 concentration initially lagged behind pD 8.4 but exhibited a distinct second phase increase, with a rapid surge after approximately 72 min of laser irradiation. The final relative Fer8 concentration was substantially higher at pD 2.0, consistent with the earlier visual observations (Figure 1).

**Figure 2:**
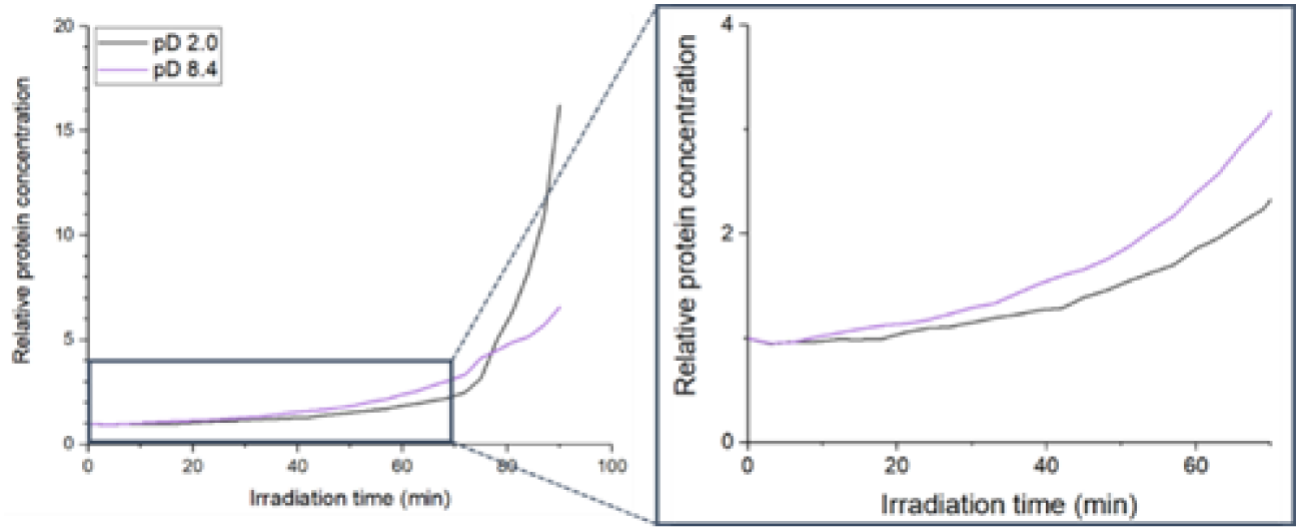
Temporal changes in relative protein concentration during laser irradiation at pD 2.0 and 8.4. The graphs depict the relative protein concentration (normalized to the initial concentration) as a function of irradiation time for solutions at pD 2.0 (black line) and pD 8.4 (violet line).

### Quantitative Analysis of Fer8 Concentration under Acidic Conditions

To investigate how the degree of acidity influences the concentration increase, we measured the time-dependent changes in relative Fer8 concentration in different acidic solutions (pD 1.5, 2.0, and 3.0). Circular dichroism (CD) spectra indicate Fer8’s existence as subunit dimers at all these pD (Figure S5).^29^ As shown in Figure 3a, solutions with higher acidity, pD 1.5 and 2.0, exhibited an apparent second phase increase in relative Fer8 concentration. This second phase increase could correspond to the initiation of amyloid fibril formation, which will be directly confirmed by subsequent ThT fluorescence and TEM analyses (see text below). In contrast, the pD 3.0 solution showed a more gradual increase pattern without a distinct second phase surge observed within 90 min of laser irradiation. This suggests that Fer8 concentration increases dynamically also under acidic conditions; thus, laser trapping behavior varies depending on the acidity.

**Figure 3:**
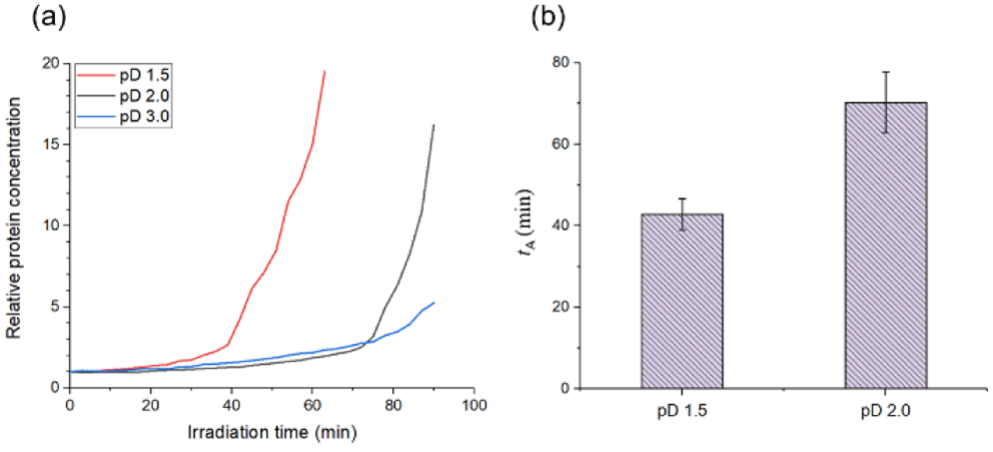
Temporal changes in relative protein concentration at the laser focus for pD 1.5, 2.0, and 3.0. (a) Relative protein concentration as a function of irradiation time. (b) The average time (*t*_A_) for the onset of the second phase of protein concentration increase at pD 1.5 and 2.0. Error bars represent standard deviations from three independent experiments.

Next, to quantitatively analyze the onset of this rapid change, we defined *t*_A_ as the time when the second phase concentration increase begins. The criteria for this analysis are detailed in Figure S6. Based on this analysis, *t*_A_ was quantitatively confirmed for pD 1.5 and 2.0 solutions. Figure 3b shows the average values and standard deviations of *t*_A_ for pD 1.5 and 2.0. The average *t*_A_ values were calculated from three independent experiments (*n* = 3) for each pD. The results showed that *t*_A_ was 40 ± 3.8 min for pD 1.5 and 67 ± 7.5 min for pD 2.0. This indicates that higher acidity conditions lead to a smaller *t*_A_, meaning the system reaches the critical concentration for amyloid fibril nucleation earlier at lower pD.

### Temporal Correlation of Protein Concentration and Amyloid Fibril Formation

To more comprehensively understand the dynamics of amyloid fibril formation, we investigate the temporal correlation between the changes in localized Fer8 concentration (observed with Dye-Fer8 fluorescence) and the concomitant formation of amyloid fibril structures, as assessed by ThT fluorescence intensity. ThT specifically binds to characteristic β-sheet structures of amyloid fibrils, causing a distinct fluorescence enhancement, thereby directly indicating amyloid formation.^35–37^ This comparative analysis helps to dissect the complex temporal relationship between protein concentration and amyloid fibril assembly.

### Temporal Changes in ThT Fluorescence Intensity

Figure 4 illustrates representative experimental results of ThT fluorescence intensity changes over time under optical trapping conditions for pD 2.0 and 8.4 solutions (Figure 4a), and for pD 1.5, 2.0, and 3.0 solutions (Figure 4b). These figures demonstrate that the ThT fluorescence intensity increase pattern varies significantly with each pD. The ThT fluorescence intensity did not increase substantially throughout the 90 min of laser irradiation in the pD 8.4 solution. This result suggests that despite optical trapping-induced concentration, amyloid fibril formation was largely hindered at pD 8.4, consistent with the monotonic increase in Fer8 concentration observed above. Conversely, in the pD 2.0 solution, a nonlinear increase in ThT fluorescence intensity was observed after 72 min of laser irradiation. This strongly suggests the formation of amyloid fibrils under laser irradiation in the pD 2.0 solution. Furthermore, Figure 4b reveals a nonlinear increase in ThT fluorescence intensity observed under all acidic pD conditions, indicating that amyloid fibrils are readily formed under acidic environments. It is also evident that the increase in ThT fluorescence intensity started earliest at pD 1.5, followed by pD 2.0, and then pD 3.0. This finding is consistent with the general understanding of amyloid fibril formation, where higher acidity promotes amyloid genesis in many proteins by favoring the unfolding of the proteins.^38–40^ A more detailed mechanism will be discussed subsequently.

**Figure 4:**
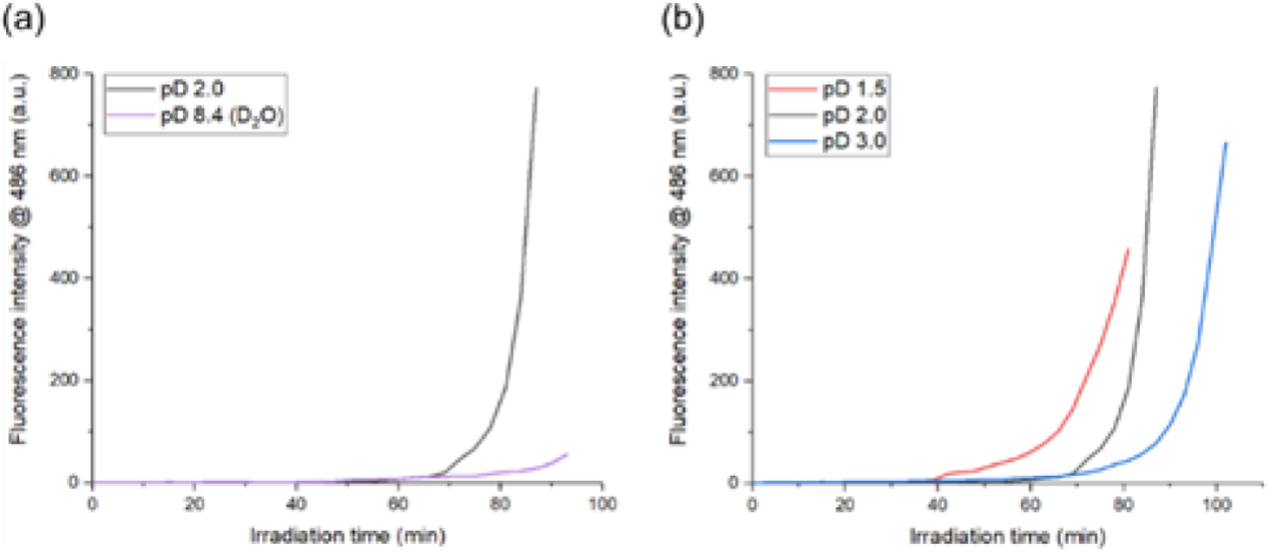
Representative temporal changes in Thioflavin T (ThT) fluorescence intensity at 486 nm (arbitrary units) under optical trapping conditions. (a) Comparison between pD 2.0 (black line) and pD 8.4 (violet line). (b) Comparison among pD 1.5 (red line), pD 2.0 (black line), and pD 3.0 (blue line).

### Quantitative Analysis of ThT Fluorescence Intensity Changes under Acidic Conditions

Here, we quantitatively analyzed the timing of the nonlinear increase in ThT fluorescence intensity under acidic conditions. As observed previously, a nonlinear increase in ThT fluorescence intensity was observed for all acidic samples. The time at which this nonlinear increase in ThT fluorescence intensity began (*t*_B_) varied with pD. Detailed methodology for determining *t*_B_ is provided in Figure S7. The average *t*_B_ values for each pD were calculated using three independent experiments (*n* = 3). Figure 5 displays the average values and standard deviations of *t*_B_ with *t*_A_ for pD 1.5, 2.0, and 3.0. This figure clearly shows that lower pD values correspond to smaller *t*_B_, indicating an earlier initiation of amyloid fibril formation. Specifically, ThT fluorescence intensity began to increase earliest in the pD 1.5 solution (average *t*_B_ = approximately 50 min), followed by pD 2.0 (average *t*_B_ = approximately 72 min), and then pD 3.0 (average *t*_B_ = approximately 80 min). These results suggest that lower pD values facilitate amyloid fibril formation under acidic conditions. This pD dependency aligns with our inference that Fer8 unfolds with acidity, promoting amyloid fibril formation at lower pD, a mechanism further discussed later in the text.

**Figure 5:**
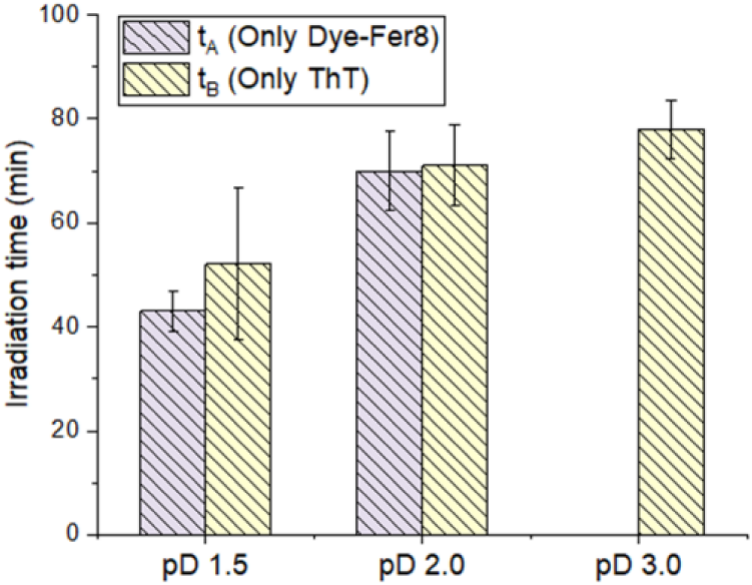
Quantitative analysis of the kinetic relationship between protein concentration and amyloid fibril formation in acidic solutions. Comparison of the time for the onset of the second phase of protein concentration increase (*t*_A_) and the time for the initiation of nonlinear ThT fluorescence increase (*t*_B_) at pD 1.5, 2.0, and 3.0.

Here, we compare the nonlinear ThT fluorescence results with the temporal changes in relative Fer8 concentration (as quantified from Dye-Fer8 fluorescence). While a slight discrepancy was observed between the average time for the onset of the second phase of protein concentration increase (*t*_A_) and the time when ThT fluorescence intensity began to increase nonlinearly (*t*_B_) at pD 1.5, these two values almost coincided for the pD 2.0 solution. This close agreement suggests a strong correlation between the second phase increase in relative Fer8 concentration and amyloid fibril formation. Specifically, it implies that the formation of dense amyloid fibrils by optically trapped and concentrated Fer8 results in a further elevation of the local protein concentration (observed with Dye-Fer8) and a sharp increase in ThT fluorescence intensity (observed with ThT).

Notably, by correlating these *t*_B_ values with the corresponding relative protein concentration observed from Dye-Fer8 fluorescence data (Figure 3a), our quantitative analysis suggested a universal critical Fer8 concentration of approximately 10–12 mg/mL (Fer8 subunit concentration, 0.53–0.63 mM) for amyloid fibril nucleation in the acidic conditions. This critical concentration appeared to be largely independent of the acidic pD. This approach is robust due to the high reproducibility of the concentration increase curves (Figure 3a) and the strong correlation between *t*_A_ and *t*_B_, which are almost identical in all pD solutions. Even for pD 3.0, where the Dye-Fer8 concentration curve did not show a distinct second phase increase, ThT fluorescence indicated amyloid formation at a similar universal critical concentration, highlighting the significance of our method in revealing these nucleation events. This strongly suggests that a specific local protein density, rather than the initial bulk acidity level, acts as a universal nucleation threshold for amyloid fibril formation in these acidic conditions. While acidity dictates the time required to reach this threshold, the concentration at which the critical nucleation event occurs appears conserved.

The potential influence of local temperature elevation due to laser irradiation in optical trapping experiments on amyloid formation must be considered. Our estimations indicate that the maximum local temperature elevation at the laser focus under our experimental conditions is approximately 2.1 K (Figure S1). This minimal temperature increase suggests that the observed amyloid fibril formation dynamics are primarily driven by the optical trapping-induced local Fer8 concentration and the consequent pD-dependent unfolding of Fer8, rather than significant thermal effects.

### Identification of Amyloid Fibrils by TEM

The fluorescence microscopy and spectroscopic measurements described earlier strongly suggest the formation of amyloid fibrils by optically trapped and concentrated Fer8 under acidic conditions. However, these results do not directly prove the presence of amyloid fibrils. Therefore, in this section, we directly confirmed the existence of amyloid fibrils by detailed structural observation of the aggregates formed by optical trapping using TEM. Figure 6 shows a representative TEM image of Fer8 aggregates formed by optical trapping in pD 1.5 solution. This image shows that the aggregates are composed of fibrous structures with a width of 4–10 nm, which is consistent with the typical dimensions of amyloid fibrils in the 7–20 nm range.^41,42^ The fibrils are some longer than 0.5 μm; however, determining the exact length of single fibrils was difficult due to their entanglement and assembly into large bundles. These fibers are consistent with the typical morphology of amyloid fibrils reported in previous studies, indicating that these aggregates are indeed amyloid fibrils. It has been reported that complete hydrolysis of proteins into very short peptide sequences is essential for forming the final apoferritin amyloid-like fibrils.^31^ However, our experiments were conducted at room temperature, where such hydrolysis is insignificant. This suggests that the intact Fer8 molecules were converted into amyloid fibrils under our optical trapping conditions.

**Figure 6:**
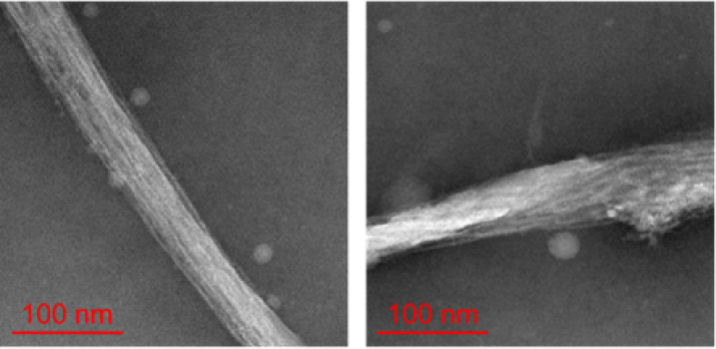
Transmission electron microscopy (TEM) image of Fer8 aggregates formed by optical trapping in pD 1.5 solution, confirming amyloid fibril formation. The image reveals distinct fibrous structures characteristic of amyloid fibrils. The scale bar represents 100 nm.

### Amyloid Fibril Formation Mechanism

This section provides an integrated discussion of the experimental results, aiming to elucidate the amyloid fibril formation mechanism of Fer8 induced by optical trapping. Specifically, we clarify how the solution’s acidity significantly influences Fer8’s folding state, the efficiency and kinetics of its concentration, and ultimately the distinct phases of amyloid fibril formation: nucleation and elongation.

Our observations at pD 8.4, where Fer8 maintains its stable 24-mer cage structure, demonstrate high initial optical trapping efficiency, leading to rapid local concentration increase. However, the absence of a significant ThT fluorescence increase under this condition (Figure 4a) suggests that this robust structure inhibits conformational changes and the subsequent nucleation event critical for amyloid fibril formation. This highlights that while concentration is significantly achieved, the intrinsic structural stability at neutral pD prevents aggregation.

Conversely, under acidic conditions (pD 1.5, 2.0, and 3.0), the initial optical trapping efficiency is reduced compared to pD 8.4, owing to the dissociation of Fer8 into subunit dimers. CD spectra under acidic conditions (pD 1.5, 2.0, and 3.0) exhibited weaker Cotton effects at 208 and 222 nm than under mildly alkaline conditions (pD 8.4), with spectral profiles consistent with those of subunit dimers (Figure S5).^29^ Furthermore, the virtual identity of the spectra across all acidic pD values suggests a consistent Fer8 dimer structure within this pD range. The dissociation of the 24-mer cage of Fer8 into its subunit dimers is permissive for amyloid formation, as evidenced by the consistent nonlinear increase in ThT fluorescence observed across all acidic pD values (Figure 4b). A key finding is that amyloid fibril formation occurred at an earlier stage under higher acidic conditions owing to the faster increase in concentration, reflected by earlier increases in both relative Fer8 concentration (*t*_A_) and ThT fluorescence (*t*_B_) at lower pD values (e.g., pD 1.5 vs 2.0 vs 3.0). This pD dependency aligns with previous reports^29^ that the structural stability of Fer8 decreases at a lower pD. This facilitated dissociation and conformational flexibility are crucial for progressing into an aggregation-prone state.

Based on our findings, we propose a mechanism of amyloid fibril formation, schematically illustrated in Figure 7. The initial stage of this mechanism involves the accumulation of Fer8 subunit dimers at the laser focus due to the optical gradient force (Figure 7a). This concentration is then followed by a critical molecular alignment step (Figure 7b). Our results suggest that the observed rapid amyloid fibril formation is likely not merely a consequence of increased local protein concentration by optical trapping. As discussed above (in the “Quantitative Analysis of ThT Fluorescence Intensity Changes under Acidic Conditions” section), the critical Fer8 subunit concentration for nucleation is estimated to be approximately 0.53–0.63 mM. To support this alignment hypothesis, we note that our recent work on lysozyme crystallization under optical trapping demonstrated that linearly polarized light significantly promotes protein crystallization through molecular alignment, while circularly polarized light does not.^33^ A similar phenomenon of polarization-dependent molecular alignment could be at play here, preorganizing Fer8 subunits into a conformation conducive to amyloid fibril formation at the laser focus. The protein’s secondary structure is maintained mainly under these acidic conditions (Figure S5). Additionally, control experiments without optical trapping further support this hypothesis (Figure S8). Even at the estimated maximum critical concentration of 0.53–0.63 mM at pD 1.5, no significant nonlinear intensity increase or notable maximum wavelength shift in ThT fluorescence was observed within a comparable time frame. Specifically, after 70 h, the intensity only reached approximately 7 times the initial, then mainly saturated for prolonged periods. This is significantly lower than the >250-fold increase observed within 81 min under optical trapping (Figure 4b), strongly suggesting that optical trapping plays a crucial role beyond simple concentration by actively accelerating the misfolding and subsequent aggregation processes. This is achieved primarily by facilitating specific molecular alignment of protein molecules due to laser polarization. Additionally, unique local environmental changes may contribute, such as increased internal pressure within the trapped condensate.

**Figure 7:**
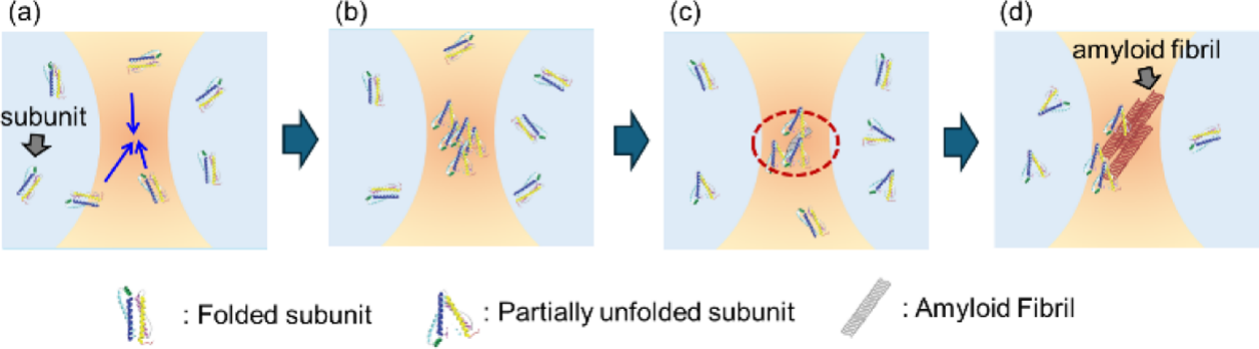
Proposed mechanism for optical trapping-induced apoferritin (Fer8) amyloid fibril formation under acidic conditions. (a) Concentration. (b) Alignment. (c) Nucleation. (d) Elongation.

This preorganization leads to the nucleation of amyloid fibrils (Figure 7c). Our quantitative analysis suggested a universal critical Fer8 concentration for amyloid fibril nucleation of approximately 10–12 mg/mL (Fer8 subunit concentration, 0.53–0.63 mM), which appeared constant irrespective of the acidic pD. This strongly suggests that a specific local protein density acts as a universal nucleation threshold for initiating the aggregation cascade under acidic conditions at all tested pD values (pD 1.5–3.0).

Finally, the nucleated fibrils undergo an elongation phase (Figure 7d), growing and extending from the trapping region. Our results also unveil a significant pD-dependence in the subsequent fibril elongation rate following this nucleation event. This is particularly evident in the distinct behavior of the pD 3.0 solution. While ThT fluorescence unequivocally confirms amyloid formation at pD 3.0 (Figure 4b), the characteristic rapid second phase increase in Dye-Fer8 concentration observed at pD 1.5 and 2.0 is notably absent. This discrepancy is critical for understanding the kinetics of amyloid formation under these conditions. At pD 3.0, Fer8 subunit dimers may be less prone to conformational changes necessary for amyloid fibril formation. At the same time, they become less stable at pD lower than 2.0 owing to the loss of the rigid tertiary structure.^29^ Therefore, while nucleation proceeds for amyloid fibril formation at a universal critical concentration for Fer8, the efficiency of fibril growth and mass accumulation is significantly diminished at pD 3.0, leading to a more gradual increase in localized concentration detected by Dye-Fer8. This interpretation underscores the complementary nature of our spectroscopic tools: Dye-Fer8 fluorescence predominantly tracks the rate of fibril formation through protein concentration, whereas ThT fluorescence confirms explicitly the presence of amyloid β-sheet structure.

## Conclusions

This study successfully demonstrated the spatiotemporal control and real-time observation of Fer8 amyloid fibril formation using optical trapping in acidic solutions. Our findings unequivocally show that Fer8 condensation behavior and amyloid fibril formation are highly dependent on the solution’s pD. While Fer8 maintained its stable 24-mer cage structure in mildly alkaline solution (pD 8.4), leading to efficient optical trapping and concentration, amyloid fibril formation was mainly inhibited. This was presumably due to the stability of the protein structure. Conversely, under acidic conditions (pD 1.5, 2.0, and 3.0), Fer8 exists as subunit dimers that are less stable than the 24-mer structure. Optical trapping-induced concentration under these conditions led to pronounced nonlinear increases in amyloid fibril formation, substantially greater than the increase in Fer8 concentration detected by Dye-Fer8 fluorescence, which clearly indicates characteristic β-sheet structures unique to amyloid fibrils. Higher acidic conditions accelerated protein condensation, with pD 1.5 exhibiting the earliest. This accelerated condensation is attributed to decreased protein stability, promoting nucleation of amyloid fibrils.

A particularly intriguing and key finding, with profound implications, is the estimation of a critical Fer8 concentration for amyloid fibril nucleation, approximately 10–12 mg/mL (Fer8 subunit concentration, 0.53–0.63 mM), which appeared constant irrespective of the solution’s acidity. This strongly suggests a universal nucleation threshold for amyloid fibril formation in these acidic conditions at all tested pD values (pD 1.5–3.0). This indicates that a specific local protein density is the fundamental requirement for initiating the aggregation cascade, regardless of environmental acidity. Given that the protein’s secondary structure is maintained mainly under these acidic conditions (as observed by CD spectra), we suggest that optical trapping may not only induce concentration but also facilitate a specific molecular alignment of Fer8 subunits at the laser focus, potentially due to the trapping laser’s polarization. This preorganization of molecules is crucial for amyloid fibril formation, leading to the observed rapid amyloid fibril formation through accelerated condensation. However, the subsequent fibril elongation rate is highly pD-dependent; the more gradual increase in Dye-Fer8 concentration at pD 3.0, despite clear ThT evidence of amyloid formation, was consistent with significantly slower fibril elongation rates compared to pD 1.5 and 2.0. This nuanced understanding highlights the distinct kinetic phases of nucleation and elongation. These results underscore the exceptional utility of optical trapping as a powerful tool for unraveling the intricate amyloid fibril formation mechanisms, providing crucial insights into early aggregation stages and potentially advancing therapeutic strategies for amyloidosis-related diseases.

## Supplementary Material



## Notes

Additional experimental details, materials, and methods, including theoretical considerations for optical trapping, calibration curves, and supplementary figures and tables (PDF)
