# Supplementary material for: Deciphering Amyloid Fibril Formation through Protein Concentration by Optical Trapping
Source: JACS Au. 2025 Oct 15;5(10):5129–36. doi: 10.1021/jacsau.5c01029 (PMC12569677; doi:10.1021/jacsau.5c01029)
Supplement: Supplementary file 1 [file au5c01029_si_001.pdf]

Supporting Information

Deciphering Amyloid Fibril Formation through Protein Concentration by Optical Trapping

Teruki Sugiyama,*[a,b] Shu-Ting Weng,[a] Tien Chen,[a] Tsuyoshi Mashima[b,c] and Shun Hirota*[b,c]

[a]Department of Applied Chemistry and Center for Emergent Functional Matter Science, National Yang Ming Chiao Tung University, No. 1001, Daxue Rd. East Dist., Hsinchu City 300093, Taiwan

[b]Division of Materials Science, Graduate School of Science and Technology, Nara Institute of Science and Technology, 8916-5 Takayama-cho, Ikoma, Nara 630-0192, Japan

[c]Medilux Research Center, Nara Institute of Science and Technology, 8916-5 Takayama-cho, Ikoma, Nara 630-0192, Japan

Table of Contents for Supporting Information

Experimental Section/Methods

- **Fer8 Purification**..S4
- **Labeling of mFluor Violet 540 Dye on Fer8**..S4
- **Preparation of Buffer Solutions with Different pD Values**..S5
- **Preparation of Fer8 Solutions with Different pD Values for Optical Trapping**............S5
- **Preparation of Fer8 and Dye-Fer8 Mixed Solution for Calibration**..............................S6
- **Preparation of Hand-Made Container**..S6
- **Optical Setup**..S7
- **CD Measurements**..S8
- **TEM Observation**..S8

SI 1. Theoretical Treatment of Optical Trapping..S9

Figures

Figure S1. Linear relationship between absorbance and optical path length for the sample solution (1.6 mg/mL Fer8, 0.025 M pD 1.5 Glycine-HCl buffer solution)..S11

Figure S2. Optical trapping and concentration dynamics of Fer8 using optical trapping..S13

Figure S3. Dependence of maximum fluorescence intensity wavelength (λ_{max}) on relative protein concentration for calibration curve generation..S14

Figure S4. Calibration curves of relative fluorescence intensity versus relative protein concentration..S15

Figure S5. CD spectra of Fer8 at pD 1.5 (red), 2.0 (black), 3.0 (blue), and 8.4 (violet)...............S16

Figure S6. Time evolution of the ratio RC_{T+3}/RC_T, used to identify the onset of the second phase of protein concentration increase..S17

Figure S7. Method for determining the onset time of ThT fluorescence nonlinear increase (t_B) and average t_B values with standard deviations for each pD condition......................................S18

Figure S8. Temporal change in fluorescence intensity at 486 nm of Thioflavin T for 12 mg/mL (0.63 mM subunit) Fer8 solutions without optical trapping..S19

Figure S9. Schematic diagram of the optical trapping setup used for real-time observation of amyloid fibril formation..S20

Tables

Table S1. Solution concentrations for the Dye-Fer8 calibration curves in pD 1.5 and pD 2.0 solutions..S21

Table S2. Solution concentrations for the Dye-Fer8 calibration curve in pD 3.0 solution..S21

Table S3. Solution concentrations for the Dye-Fer8 calibration curve in pD 8.4 solution..S21

References...S22

Experimental Section/Methods

***Fer8 Purification*:**

Recombinant horse ferritin was expressed as reported previously.[S1,S2] *Escherichia (E.) coli* Nova blue cells (Novagen) containing plasmid pKIT8, encoding the gene of horse L apoferritin without eight N-terminal residues (Fer8), were grown in LB broth at 37 °C for 24 h. The cells were harvested by centrifugation and suspended in 50 mM Tris-HCl buffer, pH 8.5, at 4 °C. After sonication with an ultrasonic liquid processor (VC 505, SONICS & MATERIALS, Inc.), the cell lysate was centrifuged to remove cell debris. The supernatant containing Fer8 was heated at 60 °C for 20 min to remove unnecessary proteins by heat denaturation and then centrifuged. The obtained supernatant was purified with a Q Sepharose anion exchange column (Cytiva) with 50 mM Tris-HCl buffer, pH 8.5, at 4 °C. Fer8 was eluted with 300 mM NaCl in the same buffer. The eluted solution was diluted with 50 mM Tris-HCl buffer, pH 8.5, at 4 °C and subsequently purified with an anion exchange column (HiTrap Q HP, Cytiva) with a 0–500 mM NaCl gradient using a fast protein liquid chromatography (FPLC) system (AKTA go, Cytiva). The absorbance was monitored at 280 nm. Subsequently, Fer8 was purified by size exclusion chromatography (SEC, HiPrep 26/600 Sephacryl S300, GE Healthcare) using the FPLC system at 4 °C with 50 mM Tris-HCl buffer, pH 8.5, containing 150 mM NaCl, and the absorbance was monitored at 280 nm. The obtained Fer8 was concentrated with an Amicon Ultra ultrafiltration tube (Merck Millipore, 100,000 NMWL), and the buffer was replaced with ultra-pure water by dialysis.

***Labeling of mFluor Violet 540 Dye on Fer8*:**

The mFluor Violet 540 dye labeling kit (AAT Bioquest) was used, which includes mFluor Violet 540 labeling dye, reaction buffer, and TQ™-dyed quench buffer. Initially, 5.0 μL of the reaction buffer was mixed with 50 μL of a 1.0 mg/mL Fer8 solution. To facilitate the reaction between the succinimidyl ester group of the labeling dye and the N-terminal of Fer8, the solution was added to a vial containing the labeling dye and incubated at room temperature for 60 minutes. The conjugation reaction was then quenched by adding 5.0 μL of quench buffer and incubating for an additional 10 minutes. The resulting labeled protein, Dye-Fer8, was subsequently diluted with 190 μL of

deuterated water (D_2O, Sigma-Aldrich, >99%) to achieve a final concentration of 0.2 mg/mL Dye-Fer8 solution, which was stored at -4 °C until use. According to AAT Bioquest, the maximum excitation wavelength for this dye is 402 nm, and the maximum emission wavelength for Dye-Fer8 ranged from 536 to 548 nm in this study, exhibiting a hypsochromic shift with increasing protein concentration (Figure S3).

***Preparation of Buffer Solutions with Different pD Values*:**

The solvent of the Fer8 solution was exchanged from H_2O to D_2O by lyophilization and re-dissolution. D_2O was chosen as the solvent instead of H_2O to prevent laser heating during optical trapping experiments, as H_2O can absorb 1064 nm laser photons due to the third harmonic of the OH stretching mode. This study used pD values instead of pH values, with pD values determined by adding 0.4 to the pH meter reading (Mettler Toledo, F20-standard)[S3]. Four different pD buffer solutions were prepared to investigate the impact of pD on Fer8 structural robustness. 37.5 mg of glycine (Sigma-Aldrich, ≥ 99%) was dissolved in 8.0 mL of D_2O for acidic buffer solutions. The pD was then adjusted to 1.5, 2.0, and 3.0, respectively, by adding appropriate amounts of a 1 M HCl D_2O solution, which was prepared by diluting 1.0 mL of 12 M hydrochloric acid (HCl, Honeywell Fluka, 37%) 12-fold with D_2O. Finally, D_2O was added to bring the total volume to 10 mL, yielding 0.05 M Glycine-HCl buffer solutions at pD 1.5, 2.0, and 3.0. For the pD 8.4 solution, D_2O was used as the sole solvent, containing 1.0 mM Tris buffer that remained from the protein purification process. The pD of this solution was measured to be 8.4.

***Preparation of Fer8 Solutions with Different pD Values for Optical Trapping*:**

Fer8 solutions at various pD values were prepared for the optical trapping experiments. The initial Fer8 solution in D_2O was prepared at a 3.2 mg/mL concentration. This concentration was determined by diluting Fer8 powder in D_2O and measuring its absorbance at 280 nm, utilizing an absorption coefficient of 14.4 $mM^{-1}cm^{-1}$. To achieve the desired pD conditions, 50 μL of this 3.2 mg/mL Fer8 stock solution in D_2O was combined with 46 μL of specific 0.05 M Glycine-HCl buffer solutions. These buffer solutions were prepared in D_2O with pD values adjusted to 1.5, 2.0, and 3.0 by adding appropriate amounts of a 1 M HCl D_2O solution. D_2O was used as the solvent

instead of H_2O to prevent laser heating during optical trapping experiments, as H2O can absorb 1064 nm laser photons due to the third harmonic of the OH stretching mode. After mixing, the pD of the solutions was carefully adjusted again using 1 M or 0.1 M HCl in D_2O as needed. Finally, D_2O was added to bring the total volume of each solution to 100 μL. For the pD 8.4 solution, D_2O was used as the sole solvent, containing 1 mM Tris buffer that remained from the protein purification process. The pD of this solution was measured to be 8.4. All prepared solutions were then filtered with a 0.22 μm syringe filter before use in the optical trapping experiments to ensure sample purity and prevent contamination.

***Preparation of Fer8 and Dye-Fer8 Mixed Solution for Calibration*:**

Calibration curves were generated to estimate the protein concentration from the Dye-Fer8 relative fluorescence intensity. For pD 1.5, 2.0, and 3.0 solutions, Fer8 aggregates that could not pass a 0.22 μm syringe filter were observed when the relative protein concentration exceeded 25 times the initial concentration. Therefore, 25× concentrated solutions were prepared by mixing 100 μL of 80 mg/mL Fer8 solution, 50 μL of 0.2 mg/mL Dye-Fer8 solution, and 50 μL of 0.1 M buffer solution. Subsequent lower concentrations for the calibration curve were obtained by serial dilution with 2.5×10^{-2} M buffer solution. The concentrations used for calibration in pD 1.5 and 2.0 are listed in Table S1, and for pD 3.0 in Table S2. For the pD 8.4 solution, the maximum relative fluorescence intensity observed in trapping experiments was 15-fold. Thus, a solution containing Fer8 at a concentration 15 times higher than the initial concentration (as indicated by Dye-Fer8 fluorescence) was prepared for calibration by mixing 100 μL of 32 mg/mL Fer8 solution, 20 μL of 0.2 mg/mL Dye-Fer8 solution, and 80 μL of D_2O. Lower concentrations of Dye-Fer8 were similarly obtained via serial dilution. The concentrations for the pD 8.4 calibration curve are presented in Table S3. Before fluorescence spectrum measurements, all solutions were filtered with a 0.22 μm syringe filter.

***Preparation of Hand-Made Container*:**

For the optical trapping experiments, a custom-made container was utilized. This

container consisted of a glued cover and a glass substrate. The glued cover was fabricated by adhering a cover glass (Matsunami, 24 mm × 24 mm, thickness 0.13–0.17 mm) to a cut-glass vial (Nichiden-Rika Glass) using silicone glue (Shin-Etsu, KE-4390). The glass substrates were prepared through a rigorous cleaning procedure to prevent contamination and enhance hydrophilicity. This involved placing ten cover glasses (Matsunami, 24 mm × 50 mm, thickness 0.13–0.17 mm) in a staining jar and sonicating them in detergent for 5 minutes. The glasses were then rinsed with pure water under sonication for 3 minutes, a step repeated three times. Subsequently, they were sonicated sequentially in acetone, pure water, and 1 M potassium hydroxide, each for 5 minutes. Finally, the glasses were rinsed again with pure water under sonication for 5 minutes, a step repeated five times. Before experiments, a cleaned glass substrate was dried and placed on the microscope stage. A 15 µL volume of the target solution was dispensed onto the highly hydrophilic glass substrate, instantly spreading to form a thin film of approximately 140–160 µm thickness. The glued cover was placed over the solution on the glass substrate to prevent evaporation during the trapping experiment.

***Optical Setup*:**

The optical setup for trapping experiments is schematically depicted in Figure S9. A near-infrared continuous-wave laser beam at 1064 nm from an Nd^{3+}: YVO_4 laser (Coherent, Matrix 1064-10-CW) was utilized as a trapping light source. After passing through a collimator composed of two convex lenses (f_1 = 100 mm and f_2 = 200 mm), the trapping laser beam was directed into an inverted microscope (IX71, Olympus). The trapping laser was focused at 20 µm above the bottom surface of the glass substrate using an Olympus 60× objective lens with a 0.90 NA. This focal position, which is well within the 140–160 µm solution thickness, ensured that the trapping occurred in an environment that was effectively a bulk solution, minimizing any potential effects from the glass-liquid or liquid-air interfaces. The trapping laser power was adjusted to 1.2 W by rotating a half-wave plate combined with a polarized beam splitter, and this power was verified using a power meter (StarLite with detector 10A-V1.1-SH, Ophir Photonics). An additional He-Ne laser (632.8 nm, Thorlabs, NHL020L) was used as an indicator to confirm the focal point of the invisible trapping laser, sharing the same optical path. A 405 nm diode visible

laser (Spectra-Physics) was employed as the excitation source for both ThT and Dye-Fer8. This excitation laser passed through a collimator (f_3 = 100 mm, f_4 = 400 mm) and then a convex lens (f_5 = 150 mm) before the objective lens to ensure broad illumination of the sample solution. The irradiated area of the 405 nm laser was estimated to be 46 μm in diameter. The 405 nm laser power was tuned to 10 μW using ND filters and confirmed with a power meter (StarLite with detector PD300, Ophir Photonics). Real-time monitoring of Fer8 trapping and aggregation dynamics was performed using a charge-coupled device camera (CCD camera, Watec, WAT-231S2) under white-light illumination from a halogen lamp. Temporal changes in fluorescence intensity during trapping laser irradiation were measured by a spectrometer (Andor Shamrock, SR-303i-A) integrated with the microscope. A 200 μm pinhole was placed before the spectrometer to collect fluorescence specifically from the focal region.

***CD Measurements*:**

Fer8 solutions at pD 1.5, 2.0, 3.0, and 8.4 were prepared by the same procedure for optical trapping experiments. CD spectra of Fer8 (subunit 14 μM) in each buffer were measured with a J-820 CD spectropolarimeter (JASCO) using a 1-mm path-length quartz cell at 25°C.

***TEM Observation*:**

For TEM observations, aggregates formed by optical trapping were recovered from the glass substrate and dispersed by sonication for 1 min. An aliquot of 2 μL of the sonicated solution was deposited on a copper grid mesh treated by a glow discharge device (JEOL HDT-400). After allowing the sample to stand for 1 min, excess liquid was removed using filter paper. The grid was rinsed with ultra-pure water and negatively stained with a 1% phosphotungstic acid solution for 1 min. The grids were scanned using a transmission electron microscope (JEM-ARM200F, JEOL) operating at 200 kV.

SI 1. Theoretical Treatment of Optical Trapping

Optical trapping is a technique that enables the non-contact trapping and manipulation of micro- and nano-sized objects in solution using the forces exerted by a focused laser beam. The fundamental principle behind optical trapping lies in the transfer of momentum from photons to the trapped object.

Optical Forces

The optical force responsible for trapping can be divided into two primary components:

Gradient Force: This force arises from the spatial gradient of the laser's intensity. It directs particles towards the region of highest intensity, which, in a focused Gaussian laser beam, is the focal point.

Scattering Force: This force is due to the momentum transfer from the photons as they are scattered by the object. It acts in the direction of the laser beam's propagation.

Rayleigh Regime

In this study, considering that the trapping targets are apoferritin (Fer8) proteins, which form 24-mer or dimeric structures, the Rayleigh theory is appropriate to describe the optical forces. The time-averaged optical force ($\boldsymbol{F}_{\mathrm{opt}}$) on the particle can be expressed as:

$$< \boldsymbol{F}_{opt} > = = \frac{1}{4} Re\{\alpha\} \nabla |\boldsymbol{E}|^2 + \sigma \frac{1}{2} Re\{\frac{1}{c} \boldsymbol{E} \times \boldsymbol{H}^*\}$$

Here, $\boldsymbol{E}$ and $\boldsymbol{H}$ are the electric field and the magnetic flux density, respectively. ∇ is the gradient operator. α is the polarizability of the particle as follows.

$$\alpha = 4\pi\varepsilon_m r^3 \frac{\varepsilon_t - \varepsilon_m}{\varepsilon_t + 2\varepsilon_m}$$

r is the particle's radius. ε_t and ε_m are the permittivity of the target particle and the surrounding medium, respectively.

The gradient force is proportional to the gradient of the electric field and the polarizability. This force directs objects with a higher refractive index than the surrounding medium towards the maximum intensity, known as the focal volume in optical trapping. The scattering force, on the other hand, acts to push the object in the direction of the Poynting vector (along the light propagation direction). The magnitude of the gradient force is proportional to r^3, indicating that optical trapping favors larger

targets. The scattering force is proportional to r^6, becoming negligible for small targets but increasing rapidly with size.

In optical trapping, a laser beam is tightly focused using a high numerical aperture (NA) objective lens. This tight focusing makes the gradient force more dominant than the scattering force, and the optical force can be approximated as:

$$< \boldsymbol{F}_{opt} > = = \frac{1}{4} Re\{\alpha\} \nabla |\boldsymbol{E}|^2]$$

Stable trapping occurs when the optical potential energy is significantly larger than the thermal kinetic energy (Brownian motion).

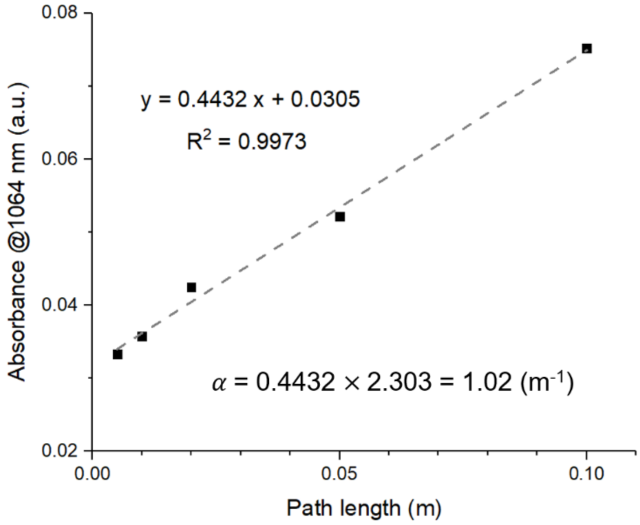

Figure S1. Linear relationship between absorbance and optical path length for the sample solution (1.6 mg/mL Fer8, 0.025 M pD 1.5 Glycine-HCl buffer solution). Measurements were performed at 1064 nm. In optical trapping experiments involving proteins, it is crucial to estimate local temperature elevation, as higher temperatures can facilitate protein misfolding. To address this, we estimated the absorbance of the sample solutions at 1064 nm, which corresponds to the wavelength of the trapping laser, according to the previous papers[S4,S5]. Initially, a Fer8 buffer solution (1.6 mg/mL Fer8, 0.025 M pD 1.5 glycine-HCl buffer solution) was placed into cuvettes with varying optical path lengths (5, 10, 20, 50, and 100 mm). The calculation of temperature elevation was performed using the Beer-Lambert law for absorption coefficient, and a relevant equation was reported. The Beer-Lambert law is given by $T = I/I_0 = e^{-\alpha l}$, where T is the transmittance, I is the input intensity, I_0 is the output intensity, α is the absorption coefficient, and l is the optical path length in meters. This equation can be rearranged to $A = -\log T = \alpha/(2.303\, l)$, where A is the absorbance, derivable from the absorption spectrum. By plotting the linear relationship between absorbance and optical path length, the absorption coefficient (α) was determined from the slope. For the sample solution, α was found to be 1.02 m^{-1} at 1064 nm. Subsequently, this value of α was used in the equation $\Delta T/\Delta P \propto \alpha/\lambda$. Assuming a thermal conductivity (λ) of 0.59, the temperature elevation coefficient ($\Delta T/\Delta P$) was estimated to be 1.73 K/W. Based on these calculations, the estimated temperature elevation in our experiments with a trapping laser power of 1.2 W was approximately 2.1 K. This minimal temperature increase suggests that the observed amyloid fibril formation

dynamics are primarily driven by the optical trapping-induced concentration and pH-dependent structural changes of Fer8, rather than significant thermal effects.

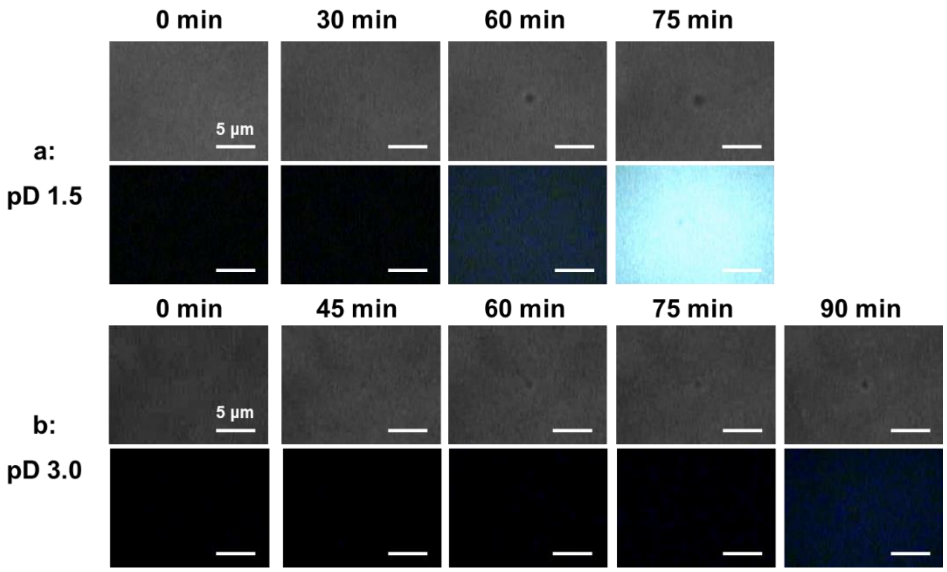

Figure S2. Optical trapping and concentration dynamics of Fer8. (a) Time-lapse transmission (top row) and Dye-Fer8 fluorescence (bottom row) images of apoferritin in pD 1.5 solution, shown from the start of laser irradiation up to 75 minutes. (b) Time-lapse transmission (top row) and Dye-Fer8 fluorescence (bottom row) images of apoferritin in pD 3.0 solution. The first image for pD 3.0 is shown at 45 minutes of laser irradiation, and images are presented up to 90 minutes. White scale bars indicate 5 µm.

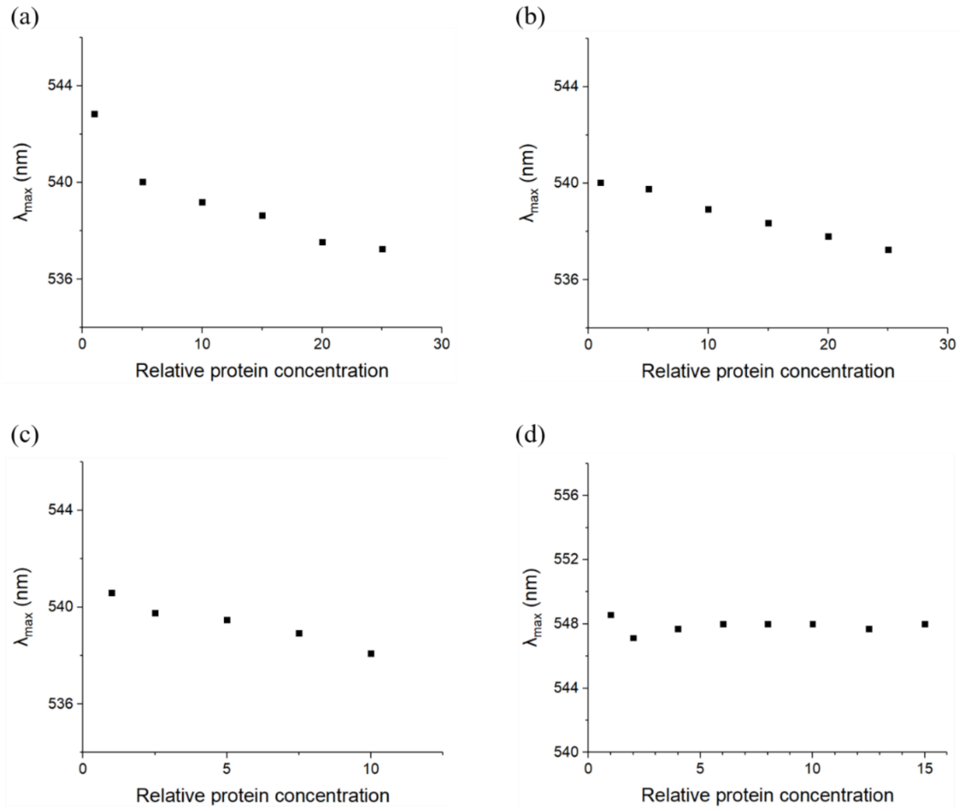

Figure S3. Dependence of maximum fluorescence intensity wavelength (λ_{max}) on relative protein concentration for calibration curve generation. (a) pD 1.5, (b) pD 2.0, (c) pD 3.0, and (d) pD 8.4 solutions. The graphs illustrate the change in λ_{max} of Dye-Fer8 fluorescence as a function of relative protein concentration. A small hypsochromic shift in λ_{max} was observed in acidic solutions (pD 1.5, 2.0, and 3.0) with increasing protein concentration, but the shift was within the resolution limit of the microscope system.

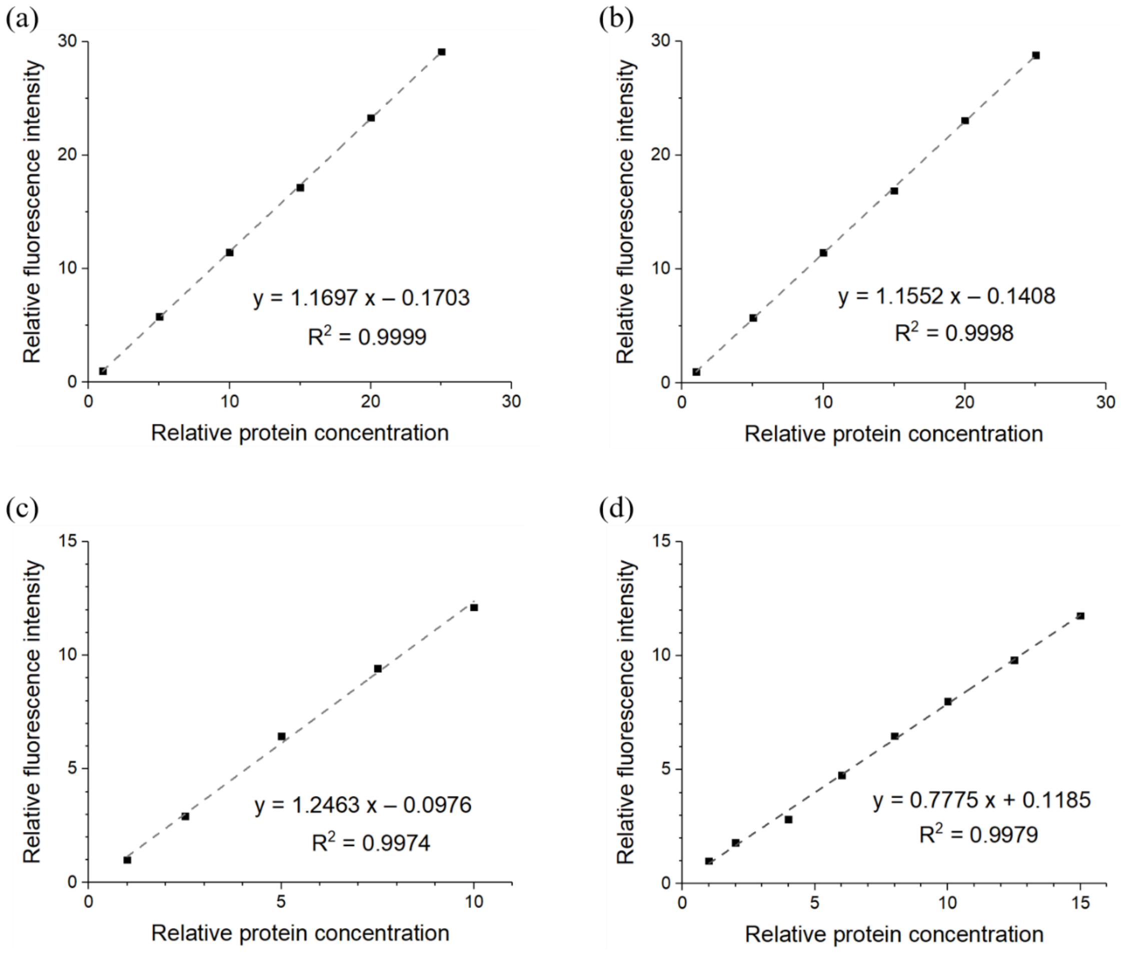

Figure S4. Calibration curves of relative fluorescence intensity versus relative protein concentration. (a) pD 1.5, (b) pD 2.0, (c) pD 3.0, and (d) pD 8.4 solutions. The graphs show the relationship between the relative fluorescence intensity of Dye-Fer8 and the relative protein concentration. Dashed lines represent the linear regression fits, quantified by the equations and coefficients of determination (R^2) shown in each graph.

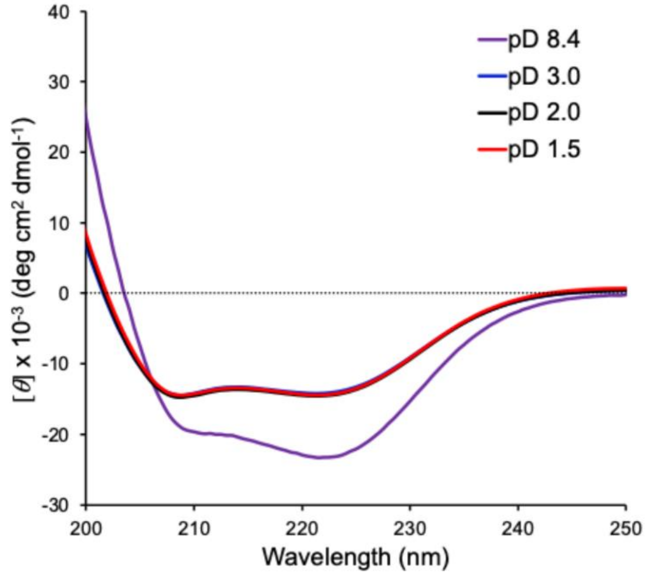

Figure S5. CD spectra of Fer8 at pD 1.5 (red), 2.0 (black), 3.0 (blue), and 8.4 (violet).

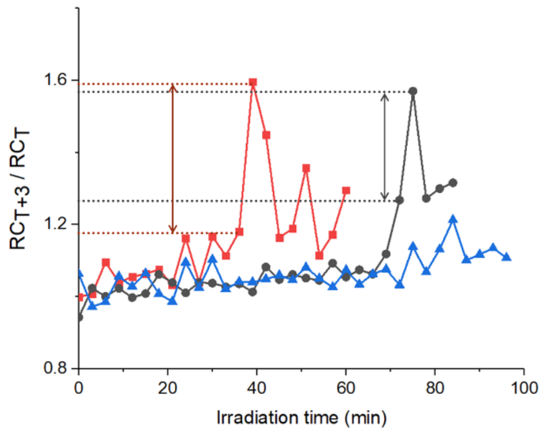

Figure S6. Time evolution of the ratio RC_{T+3}/RC_T, used to identify the onset of the second phase of protein concentration increase. The ratio of relative protein concentration, RC_{T+3}/RC_T, was calculated to evaluate the temporal change in protein concentration. RC_T and RC_{T+3} represent the relative protein concentrations at time t and t+3 min, respectively. The time point at which this ratio exhibits a sharp change is defined as t_A, indicating the start of the second phase of concentration increase. Specifically, t_A is determined as the time t when the difference in RC_{T+3}/R_{CT} values between t and t+3 min exceeds 0.2. The red line represents pD 1.5, the black line represents pD 2.0, and the blue line represents pD 3.0.

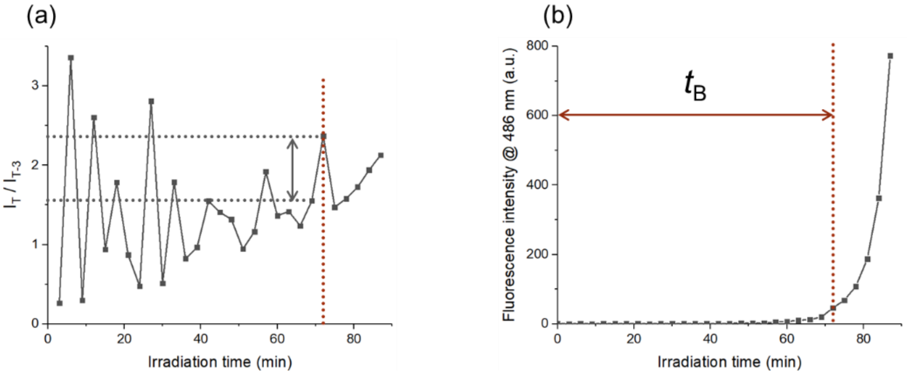

Figure S7. Method for determining the onset time of ThT fluorescence nonlinear increase (t_B) and average t_B values with standard deviations for each pD condition. (a) Time evolution of the ThT fluorescence intensity change rate (I_T/I_{T-3}). t_B is defined as the time at which the ThT fluorescence intensity change rate begins to increase non-linearly beyond a threshold value. Arrows indicate the direction of t_B determination. (b) Example of ThT fluorescence intensity change over time, with t_B determined as shown in (a). Note: In Figure S6, the ratio of relative protein concentrations was calculated using RC_{T+3}/RC_T (t and t+3 min), while in Figure S7, the ratio of ThT fluorescence intensities is calculated using I_T/I_{T-3} (t and t-3 min). This difference arises from the observation that ThT fluorescence intensity exhibits significant fluctuations at the initial stages of laser irradiation, gradually stabilizing after the onset of non-linear increase. Using I_T/I_{T-3} and referencing the fluorescence intensity at a time preceding t allows us to avoid including these initial fluctuations in the calculation and more accurately identify the point of non-linear increase. While we recognize that a unified algorithm for both metrics would be ideal for direct comparison, we chose this approach to ensure the highest accuracy in determining the onset times for each distinct fluorescence signal.

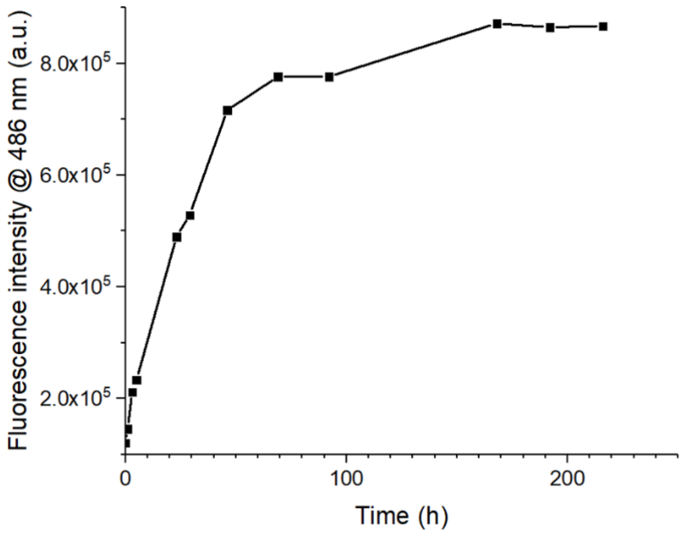

Figure S8. Temporal change in fluorescence intensity at 486 nm of Thioflavin T for 12 mg/mL (0.63 mM subunit) Fer8 solutions without optical trapping (pD 1.5).

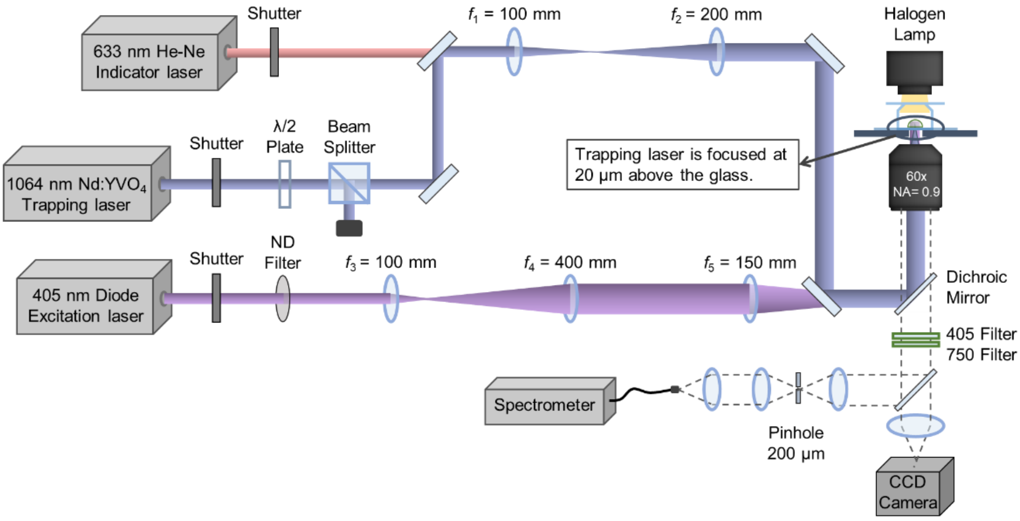

Figure S9. Schematic diagram of the optical trapping setup used for real-time observation of amyloid fibril formation.

Table S1. Solution concentrations for the Dye-Fer8 calibration curves in pD 1.5 and pD 2.0 solutions.

Relative concentration	1	5	10	15	20	25
Fer8 concentration (mg/mL)	1.6	8	16	24	32	40
Dye-Fer8 concentration (10^{-3} mg/mL)	2	10	20	30	40	50
Buffer concentration (10^{-2} M)			2.5			

Table S2. Solution concentrations for the Dye-Fer8 calibration curve in pD 3.0 solution.

Relative concentration	1	2.5	5	7.5	10
Fer8 concentration (mg/mL)	1.6	4	8	12	16
Dye-Fer8 concentration (10^{-3} mg/mL)	2	5	10	15	20
Buffer concentration (10^{-2} M)			2.5		

Table S3. Solution concentrations for the Dye-Fer8 calibration curve in pD 8.4 solution.

Relative concentration	1	2	4	6	8	10	12.5	15
Fer8 concentration (mg/mL)	1.6	3.2	6.4	9.6	13	16	20	24
Dye-Fer8 concentration (10^{-3} mg/mL)	2	4	8	12	16	20	25	30

References

[S1] M. Yamanaka, T. Mashima, M. Ogihara, M. Okamoto, T. Uchihashi, S. Hirota, *PLoS One*, **2021**, *16*, e0259052.

[S2] K. Yoshizawa, Y. Mishima, S.-Y. Park, J. G. Heddle, J. R. H. Tame, K. Iwahori, M. Kobayashi, I. Yamashita, *J. Biochem.*, **2007**, *142*, 707–713.

[S3] K. Mikkelsen, S. O. Nielsen, *J. Phys. Chem.*, **1960**, *64*, 632–637.

[S4] K. Setoura, K. Fujita, S. Ito, *J. Nanophotonics* **2018**, *13*, 1.

[S5] S. Ito, T. Sugiyama, N. Toitani, G. Katayama, H. Miyasaka, *J. Phys. Chem. B* **2007**, *111*, 2365–2371.
